# The effectiveness of national guidance in changing analgesic prescribing in primary care from 2002 to 2009: An observational database study

**DOI:** 10.1002/j.1532-2149.2012.00189.x

**Published:** 2012-07-02

**Authors:** J Bedson, J Belcher, OI Martino, M Ndlovu, T Rathod, K Walters, KM Dunn, KP Jordan

**Affiliations:** 1 Arthritis Research UK Primary Care Centre, Primary Care Sciences, Keele UniversityStaffordshire, UK; 2 Department of Public Health, Epidemiology and Biostatistics, University of BirminghamUK; 3 UCL Research Department of Primary Care and Population HealthLondon, UK

## Abstract

**Background:**

Numerous national guidelines have been issued to assist general practitioners’ safe analgesic prescribing. Their effectiveness is unclear. The objective of this study was to examine trends in general practitioners’ prescribing behaviour in relation to national guidelines.

**Methods:**

This was a retrospective observational database study of registered adult patients prescribed an analgesic (2002–2009) from the Consultations in Primary Care Archive – 12 North Staffordshire general practices. Prescribing guidance from the UK Medicines Regulatory Health Authority (MHRA) regarding non-steroidal anti-inflammatory drugs (NSAIDs) and co-proxamol, and the National Institute for Health and Clinical Excellence (NICE) osteoarthritis (OA) management guidelines were considered. Analgesic prescribing rates were examined, arranged according to a classification of six equipotent medication groups: (1) basic analgesics; (2)–(5) increasingly potent opioids and (6) NSAIDs. In each quarter from 2002 to 2009, the number of patients per 10,000 registered population receiving a prescription for the first time from each group was determined. Quarters associated with significant changes in the underlying prescribing trend were determined using joinpoint regression.

**Results:**

A significant decrease in incident co-proxamol and Cox-2 prescribing occurred around the time of the first MHRA advice to stop using them and were rarely prescribed thereafter. The new prescribing of weak analgesics (e.g., co-codamol 8/500) increased at this same time. Initiating topical NSAIDs significantly increased around the time of the NICE OA guidelines.

**Conclusions:**

Significant prescribing changes occurred when national advice and guidelines were issued. The effectiveness of this advice may vary depending upon the content and method of dissemination. Further evaluation of the optimal methods for delivering prescribing guidance is required.

## 1. Introduction

General practitioners (GPs) are subjected to external influences that might change their prescribing behaviour. These include research publications, pharmaceutical representatives and local medication formularies (NHS Stoke on Trent, 2010). Additionally, there are prescribing guidelines from the National Institute for Health and Clinical Excellence (NICE, 2010a), or advice from the Medicines and Healthcare products Regulatory Agency (MHRA) in the United Kingdom informing GPs of ‘preferred’ or ‘recommended’ prescribing. However, the individual GP makes any final prescribing decision and national guidance may have little influence on this. Previous studies have shown poor concordance between GP prescribing and national guidance in areas such as statin prescribing (Mantel-Teeuwisse et al., 2006). Printed educational materials have shown only a marginal level of effectiveness in changing doctors’ practice (Farmer et al., 2008; Wensing et al., 2010). Conversely, Swedish guidelines were effective in implementing the prescribing of disease-modifying anti-rheumatic drugs (Carli et al., 2008), while in the United States, Food and Drug Administration Advisory guidance accelerated the decline in use of antipsychotic drugs in dementia (Kales et al., 2011). National guidelines include targeted and specific advice from the MHRA on prescribing of individual medications in relation to drug safety, or general advice on the management of a disease, such as that issued by NICE. The effectiveness of these different types of prescribing guidelines is currently uncertain.

**What's already known about this topic?**

- The effectiveness of national guidelines on modifying prescribing behaviour has been inconsistent.
- Analgesics are among the commonest prescribed medications in primary care.
- From 2002 to 2009, several advisory guidelines on analgesic prescribing have been issued. It is unknown how effective this guidance has been.

**What does this study add?**

- Directive guidance on drug safety issues appears most effective in changing prescribing behaviour.
- Evidence-based national guidelines have a more limited benefit.

Analgesics are commonly prescribed in the United Kingdom. One study found 10% of all prescriptions in 1 year were for painkillers (Bedson et al., 2010). The overall 4-week prevalence for any pain symptom has been estimated at 66% in the general population (Gureje et al., 1998). Additionally, over €12 billion of lost employment productivity is related to musculoskeletal pain highlighting its economic impact (Bevan et al., 2009).

The MHRA is required to safeguard public health by ensuring that medicines are acceptably safe. Since 2004, GPs have been informed by the MHRA of several evidence-based reviews relating to adverse analgesic events (MHRA, 2004, 2005a,b, 2006). One related to the toxicity of co-proxamol in overdose (MHRA, 2005b), others advised against the use of certain non-steroidal anti-inflammatory drugs (NSAIDs) due to an apparent increase in the risk for certain patients of myocardial infarction (MHRA, 2004). The NICE osteoarthritis (OA) guidelines attempted to rationalize prescribing for this condition (NICE, 2008). NICE advocated the earlier use of topical NSAIDs, and the appropriate use of oral NSAID medications in tailored groups of patients (NICE, 2008). This was a reversal of guidance from a previous review of NSAIDs by the MHRA in 2004 (MHRA, 2004).

We hypothesized that the national guidance such as that issued by NICE would be more likely to have a major impact on prescribing due to its prominence and construct i.e., based on the best evidence. This study's objectives, therefore, were to investigate trends in analgesic prescribing from 2002 to 2009, a period during which the MHRA and NICE issued advice regarding analgesic prescribing, and determine how prescribing changes related to the different types of guidance given to GPs.

## 2. Methods

The study employed the Prescriptions in Primary Care Archive (PiPCA). PiPCA is a part of the Consultations in Primary Care Archive (CiPCA). In CiPCA, anonymized primary care data has been remotely extracted from 12 general practices within the Keele GP Research Partnership (Porcheret et al., 2004). Ethical approval for CiPCA was granted by the North Staffordshire Research Ethics Committee for epidemiological research. The practices have a research agreement with Keele and code clinical activity to a high standard having followed the Keele consultation data audit, training and validation programme (Porcheret et al., 2004). The quality of the data is comparable to, or better than, that of larger national general practice databases (Jordan et al., 2007). Prescribing and demographic data from the 12 practices that have contributed to PiPCA continuously from 2001 to 2009 were analysed for people aged 15 and over. These practices are from a mix of more and less deprived areas.

**Figure 1:**
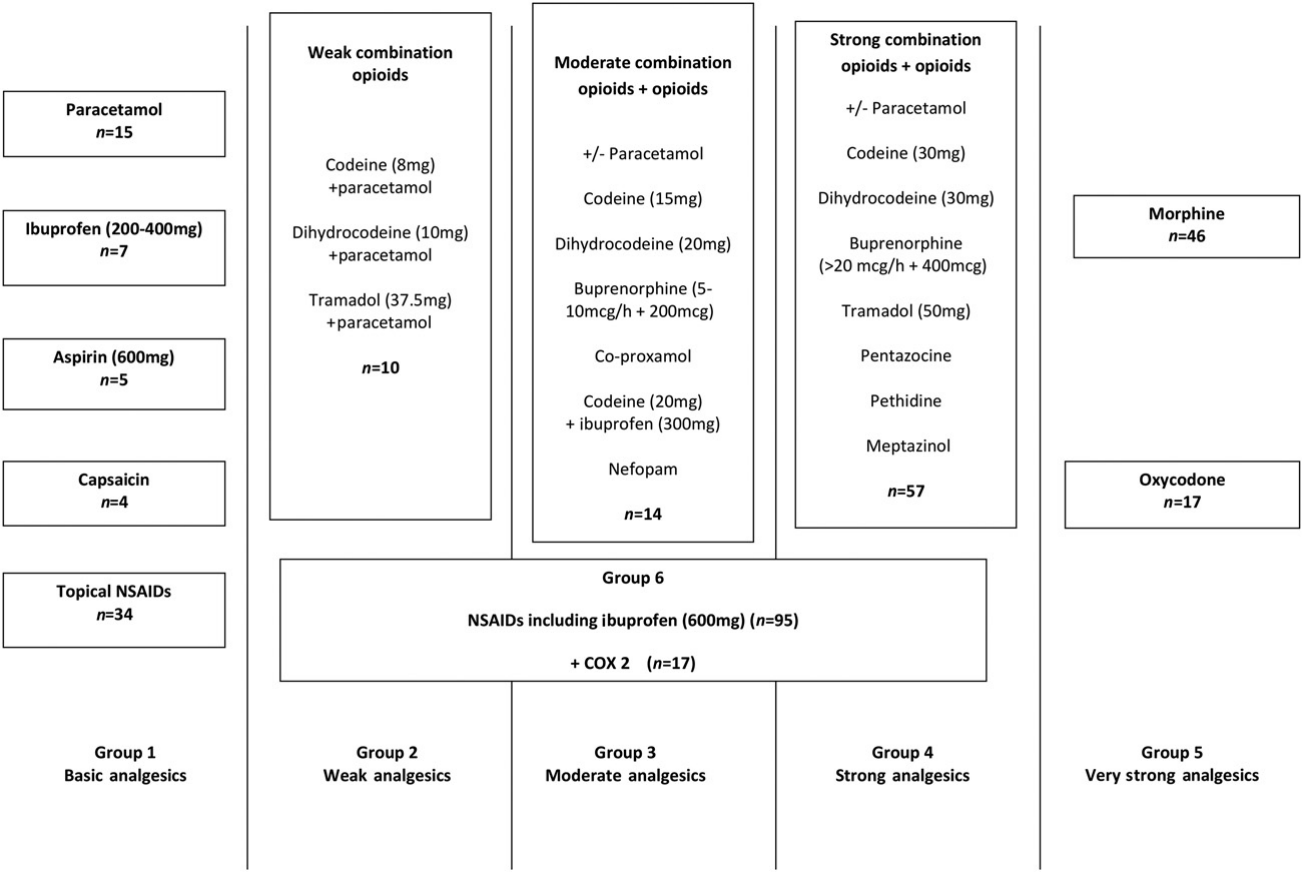
Analgesic categorization for prescribing analgesics and NSAIDs in primary care where *n* = number of individual prescribable medication within that group.

There are in excess of 300 separate analgesic preparations listed within the British National Formulary (BNF, 2011). It was therefore, considered necessary to rationalize all analgesics within the BNF into separate groups of medications, which were considered equally effective when treating a given level of pain. These were then arranged hierarchically according to increasing analgesic potency. A four-step consensus exercise was undertaken with five academic and 20 practising GPs using nominal group and Delphi consensus methods (full methodology available from the authors) to construct the categorization of analgesics. The final categorization consisted of six groups, group 1 (basic analgesics including paracetamol), groups 2–4 (increasingly strong opioids used alone or in combination with paracetamol), group 5 (very strong single opioids such as morphine) and group 6 (NSAIDs), which were considered separate to groups 1–5 in terms of analgesic potency (Fig. 1). This model did not include medications used in the treatment of neuropathic pain in order maintain consistency with existing models such as the NICE OA guidelines (NICE, 2008) and the World Health Organization analgesic ladder (Ehrlich, 2003), which currently do not incorporate these medications.

Changes in GP prescribing behaviour in relation to six major pieces of national guidance were analysed. As it is difficult to demonstrate the individual effects of interventions grouped closely together in time, those within 3 months of each other were grouped together and considered as a single intervention. The interventions considered in the study are shown in Table 1. Advice issued by the MHRA is sent on an individualized basis using personal letters to all prescribing doctors ensuring that all GPs are aware of the changes suggested in analgesic use. NICE also disseminate their guidelines to specific groups that include all GPs. (NICE, 2010b).

**Table 1:** Intervention groupings in relation to their time of issue

Intervention	Date	Content
1	21 December 2004	MHRA. Stop using Cox-2 in patients with IHD. Use lowest dose NSAID (MHRA, 2004).
	21 January 2005	MHRA. Withdraw co-proxamol over next 3 years (MHRA, 2005b).
	17 February 2005	MHRA. Cox- 2 should not be used in heart disease (MHRA, 2005a).
2	August 2005	MHRA. Use lowest dose NSAID tailored to individual risk profile (MHRA, 2005c).
3	October 2006	MHRA. Non-selective NSAIDs may be associated with a small increased risk of thrombotic events (MHRA, 2006).
4	February 2008	NICE OA management guidelines issued (NICE, 2008).

### 2.1 Annual prescription prevalence

All adults aged 15 and over with a recorded prescription for a pain medication from 2001 to 2009 were identified. Then, annual prescription prevalence was calculated per 10,000 registered population for each analgesic group, with 95% confidence interval. The numerators for calculating annual prescription prevalence were the number of patients receiving at least one prescription within a group within each calendar year. Repeat or multiple prescriptions in the same group following the first prescription for a medication in that group in each year were ignored. The denominators were the mid-year adult population registration figures over the 12 registered practices for each year. The total registered population aged 15 and over of the practices ranged from 84,064 (2005) to 88,069 (2008) but the age/sex distribution did not vary. Across all years, 22% were aged 65 and over and 51% were female. Hence, it was not felt necessary to standardize the prevalence figures.

### 2.2 Effect of interventions on new prescribing

Each year was divided into quarterly time periods and the number of people prescribed at least one new analgesic within an analgesic group was extracted for each time period from 2002 to 2009. A new analgesic was defined as no previous analgesic prescription issued from within the same group in the previous 12 months. The quarters were defined on a seasonal basis from the second quarter of 2002 (comprising March, April and May) to the last quarter of 2009 (September, October and November); for easy distinction, the quarters are labelled 2002q2 to 2009q4. The Quality and Outcomes Framework contained in the new General Medical Services contract for GPs in the United Kingdom requires GPs to review their patient's repeat prescriptions on an annual basis (Department of Health, 2012). Consequently, we have considered January 2001 to February 2002 as a run in period for the study since this then decreases the prospect of including repeat prescriptions in the analysis. The quarterly number of people per 10,000 registered population receiving a drug in each group who had not received a drug from this group in the 12 months prior to that quarter was determined. The denominators were the mid-year adult populations. In view of the specific nature of the interventions relating particularly to Cox-2 inhibitors (intervention 1), topical NSAIDs (intervention 4) and co-proxamol (intervention 1), these subgroups were also analysed separately.

The four interventions (Table 1) occurred at time points 2005 quarter 1 (q1), 2005q3, 2006q4 and 2008q1 splitting the 8 years into five segments.

### 2.3 Statistical analysis

Joinpoint regression was used to identify quarters where a marked change (the ‘joinpoint’) in the underlying trend in the incidence of new prescriptions occurred for each group of analgesia (Kim et al., 2000; Fay et al., 2006; National Cancer Institute, 2011). Permutation tests using Monte Carlo methods were used to determine the minimum number of joinpoints required to provide an adequate fit to the data. A significance level of 1% was used to assess the need for each extra joinpoint, starting from zero joinpoints. The final model consists of a series of linear lines with different slopes connected together at the joinpoints. The time point for the start of each identified change in the underlying prescribing trend (the joinpoint) can then be compared with the dates of the interventions. If no joinpoints were identified, this would indicate no significant change in the underlying trend in prescribing between 2002 and 2009. Models were fitted using the joinpoint regression program (National Cancer Institute, 2011).

## 3. Results

### 3.1 Annual prescription prevalence

Annual prescription prevalences between 2001 and 2009 are given in Supporting Information Table S1. The annual prescription prevalence of all analgesics remained stable at around 3100 patients prescribed at least 1 analgesic per 10,000 registered population annually. Prescribing of moderate analgesics fell by two-thirds from 754/10,000 in 2001 to 230/10,000 in 2006 before increasing to 287/10,000 in 2009. Co-proxamol prescriptions fell from 736/10,000 in 2001 to 11/10,000 in 2009. Prevalence of Cox-2 inhibitor prescribing more than doubled from 167 patients/10,000 in 2001 to 378/10,000 in 2004 but then fell to 65/10,000 by 2009. By contrast, the number of people prescribed topical NSAIDs more than doubled over the time period (from 272/10,000 in 2001 to 602/10,000 in 2009). Prescribing of strong analgesics (507/10,000 to 955/10,000) and very strong analgesics (38/10,000 to 74/10,000) almost doubled from 2001 to 2009. There were smaller increases for basic and weak analgesics.

### 3.2 Effect of interventions on new analgesic prescribing

**Figure 2:**
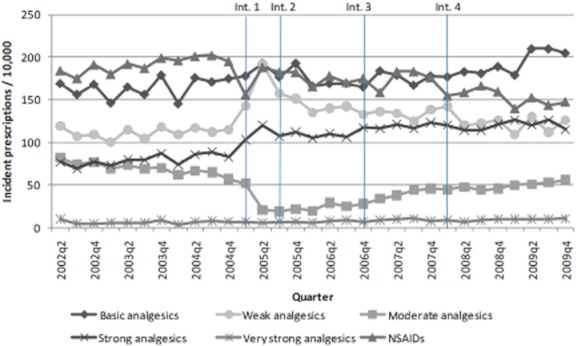
Incident number of patients per 10,000 registered population prescribed an analgesic from the six groups per quarter.

Fig. 2 shows the trends in new analgesic prescribing over the period 2002–2009 for each of the six main groups of analgesia. Particularly noticeable are the sharp fall in number of people newly prescribed moderate analgesics and the decline in NSAID prescribing around the time of intervention 1 with increases in new weak and strong analgesic prescribing.

**Figure 3:**
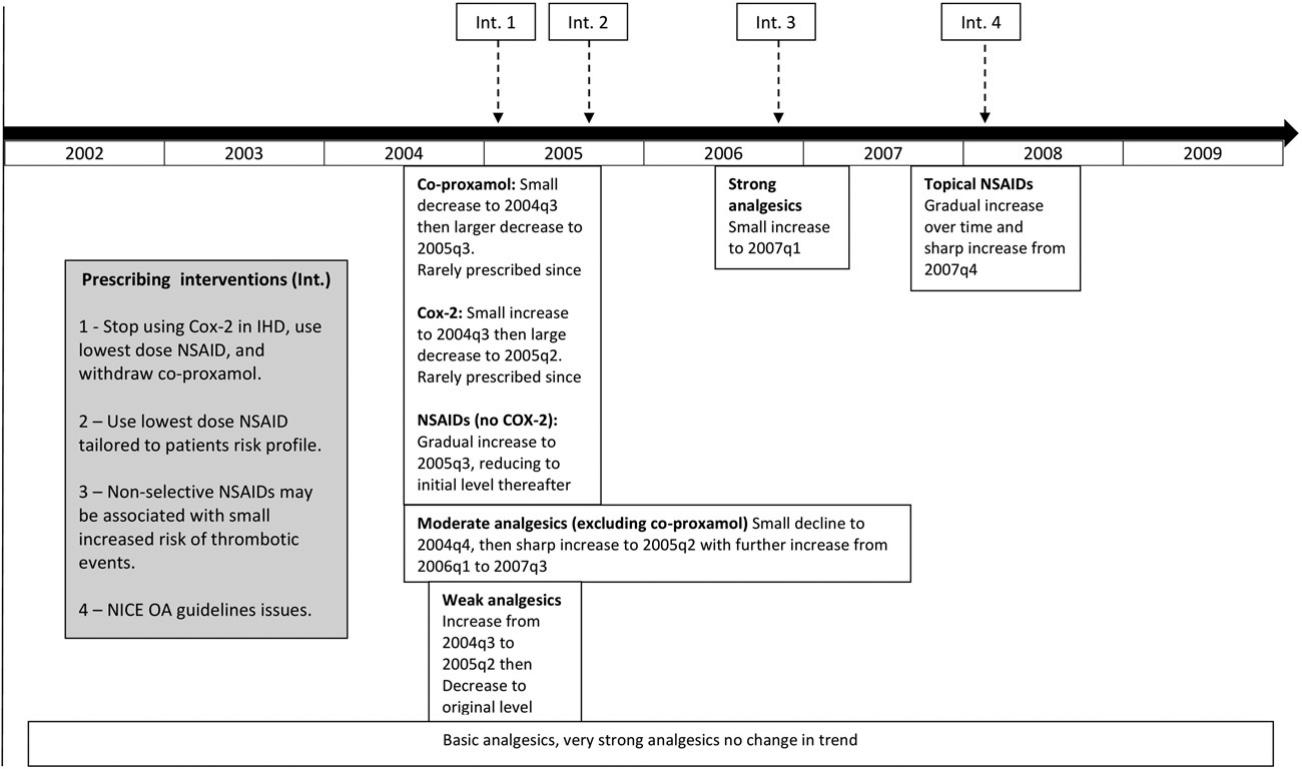
Significant changes in trends in new analgesic prescribing and time of national prescribing interventions 2002–2009.

Fig. 3 illustrates the temporal relationship between significant joinpoint quarters, changes in analgesic prescribing and national prescribing interventions from 2002 to 2009. Detailed results of the joinpoint regression analyses are given in Supporting Information Tables S2 and S3.

**Figure 4:**
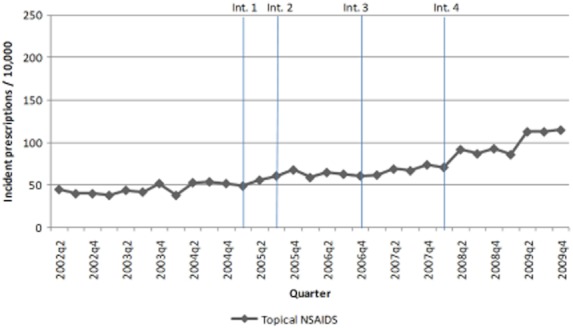
Incident number of patients per 10,000 registered population prescribed topical NSAIDs per quarter.

No joinpoints were identified for basic analgesics or very strong analgesics, which both had a small rise in prescribing over the 9 years in their underlying trend. However, topical NSAIDs, a basic analgesic, did increase over time with a sharper increase shown starting from the quarter (2007q4) before the launch of the NICE OA guidelines (intervention 4; Fig. 4).

**Figure 5:**
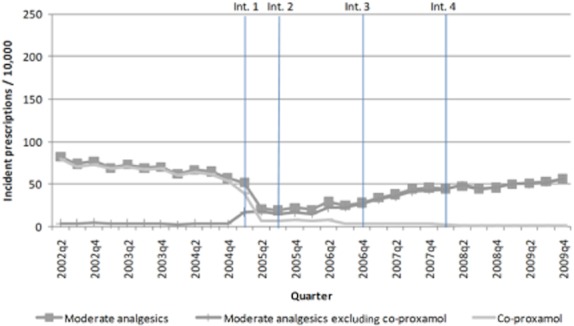
Incident number of patients per 10,000 registered population prescribed moderate analgesics per quarter.

**Figure 6:**
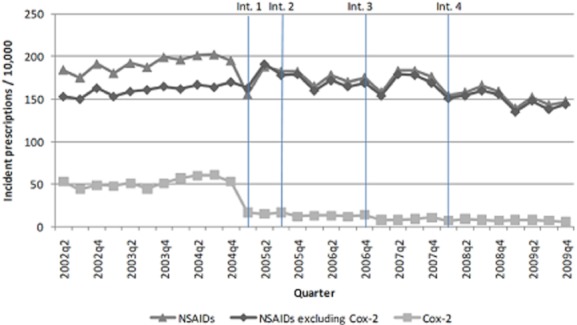
Incident number of patients per 10,000 registered population prescribed NSAIDs per quarter.

The quarters immediately prior to intervention 1 coincided with significant joinpoints indicating sharp declines in new all moderate analgesic prescribing, particularly co-proxamol (from 2004q3; Fig. 5). Cox-2 inhibitors also showed the start of a dramatically significant decrease in new prescribing around this time (Fig. 6). By contrast, new weak analgesic prescribing and moderate analgesic prescribing other than for co-proxamol started to significantly increase at this time. These trends all lasted around 9 months to the period just after intervention 1, which was also around the time of intervention 2 (2005q2, 2005q3). By this time, co-proxamol and Cox-2 inhibitors had become very infrequently used as new prescriptions. Co-proxamol had a quarterly prescription incidence of 1/10,000 registered population from 2008 while new prescribing of Cox-2 inhibitors stabilized at around 8/10,000 by 2007.

The increase in new prescriptions for weak analgesics did not last. By 2009, incident prescriptions for weak analgesics were similar to their 2002 levels. However, new prescribing of moderate analgesics excluding co-proxamol started increasing again from 2006q1.

NSAIDs prescribing excluding Cox-2 inhibitors had one identified joinpoint with prescribing incidence increasing gradually up to the time of the second intervention (which covered use of low dose NSAIDs) before falling to initial levels. Prescribing of strong opioid combinations showed a generally increasing trend from baseline, which flattened out around the one joinpoint identified 2007q1.

## 4. Discussion

This study demonstrated dramatic reductions in new prescribing of moderate analgesics (especially co-proxamol) and Cox-2 anti-inflammatory, balanced by increases in weak analgesic prescribing in the 6 months prior to related specific prescribing guidance from the MHRA. At the time of the release of the NICE OA guidelines, there was an increase in the prescribing of topical NSAIDs. Overall prescribing (repeat plus new) also followed these trends as shown by the annual prescription prevalence figures.

MHRA initiatives include direct communiqués with the GP regarding specific drug safety issues, supplemented by press releases and media attention. Locality-based Primary Care Trust (PCT) prescribing advisors reinforce these safety messages to doctors (Braybrook and Walker, 2000). This ‘cascade’ method may potentially be more effective in delivering the ‘message’ to GPs. This is exemplified by the MHRA guidance on co-proxamol and Cox-2 prescribing. In the case of co-proxamol, the MHRA recommended phasing out its use (intervention 1). The changes in prescribing trends that started in the period before, and then became far more substantial during the time period when guidance on the use of these mediations was being issued suggests that the changes in prescribing were related to the guidance (Braybrook and Walker, 2000). The changes prior to the issuance of guidance are likely to be related to attention on the research findings regarding potential adverse events that would have been available in the medical media, and these were subsequently reinforced by the MHRA guidance. A decrease in co-proxamol use at this point was also reported in a previous study as well as similar changes in trends for opioid analgesics such as co-codamol, co-dydramol and codeine (weak analgesics; Hawton et al., 2009). The NHS Business Authority report for PCT prescribing showed a similar decreasing trend in the use of Cox-2 usage during this period (NHS Business Authority, 2011). This suggests that our findings are reflective of prescribing at a national level. Switching to alternative analgesics appears to have occurred. A sharp increase in weak analgesic use occurred in the same time frame as the reduction in Cox-2 prescribing and co-proxamol. Subsequently, when weak analgesic use decreased, an increase in moderate analgesics (excluding co-proxamol) occurred, which may be explained by alternative moderate analgesia to co-proxamol starting to be used. Additionally, with the reduction in Cox-2 use, NSAIDs (excluding Cox-2) showed a slight increase before falling back gradually to initial levels.

Intervention 4 relates to the NICE OA guidelines. Prior to these guidelines, there had been debate about the effectiveness of topical NSAIDs (Bandolier, 2011), but the main effect appeared to be their increased use. Other advice related to using opioids (as moderate, strong or very strong analgesics) or oral NSAIDs (including Cox-2s) as a third line of therapy, but no change in their use was seen.

It is possible that NICE guidelines, a summation of best evidence relating to managing the disease process in question, are issued at a time when this best evidence pertaining to the condition has already been enacted by GPs following publication of individual trial evidence, systematic reviews or specific prescribing guidance from regulatory bodies such as the MHRA. The change in topical NSAID use may have occurred simply as the result of a ‘green light’ effect, whereby NICE were lending credence to do more of what with what was already happening. Previous studies have identified the limited benefit of guidelines (Farmer et al., 2008), while others have shown that guidance with multifaceted aspects, incorporating social influence and management support can be substantially more effective (Braybrook and Walker, 2000; Wensing et al., 2010). NICE OA guidelines relate to managing pain in osteoarthritis. We have examined the prescribing rates of all general analgesics (excluding medications used in neuropathic pain), regardless of the indication. These may be used for pain relating to conditions other than OA. Consequently, the NICE OA guidelines might appear less effective than if we had only used a population suffering with OA-related pain, although in general, musculoskeletal conditions do account for the majority of consultations for pain in primary care (Jordan et al., 2010). Prescribing habits may be more profoundly influenced when the emphasis of the message relates to ‘risk’ and ‘adverse outcomes’. Doctors are increasingly ‘risk’ aware (MDDUS, 2011), and it is probable that communications relating to a serious harmful outcome will be acted upon. When advice, such as that issued by NICE, focuses more on prescribing that is designed to ensure a favourable outcome, it may be that the message carries less gravitas since not following the advice will not necessarily end in life-threatening consequences for the patient.

Our study reports on the temporal associations of changes in prescribing practices with the release of national prescribing guidance and changes in prescribing may have been influenced by other factors, such as publication of related evidence or production of local guidelines. For example, Hawton published findings on the associated risks of using co-proxamol and accidental poisoning in 2003; however, this was 2 years before the MHRA guidance on its use was issued (Hawton et al., 2003). Local guidelines on analgesic use were issued in North Staffordshire as well, but this was 12 months following the MHRA guidance and did not include advice on co-proxamol or NSAID use and do not appear to have changed underlying trends in prescribing (New Medications Committee, 2006).

A possible limitation of this study is the inclusion of practices from one area of the United Kingdom, which is slightly more deprived than England as a whole. The categorization of analgesics into six groups developed to rationalize the analysis was undertaken by a rigorous consensus process using practicing GPs and may include misclassification of drugs into equipotent groups. However, the similarity of some of our results with previous work that examined national prescribing rates indicates that our findings are likely to be representative of the population in general (Hawton et al., 2009). We have been unable to examine the level of analgesics bought over the counter (OTC) by patients when the changes examined within this study took place. In the case of basic analgesics and weak combination opioids, the rates provided are likely to be an underestimate of the number of people actually taking these drugs, particularly as they are often cheaper to purchase OTC for patients who pay for prescriptions. However, since the amount of medication that can be bought OTC is limited by regulation, in those with chronic pain, it may be more cost-effective to obtain analgesia on prescription (MHRA, 2009). In addition, prescriptions for those aged over 60 years and on low incomes in the United Kingdom are free. The findings of our study would appear to support this in that following discontinuation of co-proxamol, there was an increase in weak analgesics prescriptions, which contain those drugs that might be bought OTC. We did not analyse other clinical information in the database to interrogate the appropriateness of analgesics prescription in the context of the MHRA and NICE guidance, rather in this study, we have undertaken a population level of assessment in trends, which have assessed how general use has changed following the directives and intervention. The changes identified do suggest that GPs are considering appropriate use of medications. Whether the appropriateness has improved is an area for further research but is very complex as it involves many factors, not just reason for consultation, but also including concurrent comorbidity, previous OTC and concomitant prescribed medication, patient preference etc., which would have been difficult to evaluate in this study.

The guidelines and advice assessed in this study address individual drugs and groups. In recognition of this, we examined specific drugs when the guidance related to that particular drug (e.g., co-proxamol) or group (e.g., cox-2 NSAIDs). However, to examine the secondary effect of this on other analgesic prescribing, we used the consensus groups to observe prescribing trends in terms of drugs GPs might use to substitute for an equivalent analgesic effect. In particular, did they use a ‘like for like’ analgesic or change to a lesser or more potent painkiller. The consensus model was more suited to this investigation. Additionally, the consensus exercise recognized that NSAIDs have an anti-inflammatory element, so they could not fit into another analgesic group easily, and often are used in conjunction with other analgesics where GPs co-prescribe them.

Our findings suggest that significant changes in prescribing occur at times when national advice and guidelines are issued to GPs. This is promising in that the advice would appear to have had the desired effect, although the level of effectiveness may vary depending upon the content of, and method of dissemination of the information in primary care. Additionally, future investigation of the relationship between analgesic prescribing and medications for comorbid disease such as cardiovascular or respiratory problems would also help further understand this complex area of prescribing. Further study of national guidelines effectiveness in relation to other medications is required to evaluate the best methods for delivering prescribing guidance in order to affect positive change in professional behaviour.

## Supporting Information

**Table S1.** Annual prescription prevalence per 10,000 registered patients 2001–2009 (95% confidence interval).

**Table S2.** Joinpoint regression results: changes in underlying trend in incidence of prescribing for basic, weak and moderate analgesics.

**Table S3.** Joinpoint regression results: changes in underlying trend in incidence of prescribing for strong and very strong analgesics and NSAIDs.
