# Supplementary material for: The effectiveness of national guidance in changing analgesic prescribing in primary care from 2002 to 2009: An observational database study
Source: Eur J Pain. 2012 Jul 2;17(3):434–43. doi: 10.1002/j.1532-2149.2012.00189.x (PMC3592995; doi:10.1002/j.1532-2149.2012.00189.x)
Supplement: Supplementary file 1 [file ejp0017-0434-SD1.pdf]

table S1: Annual prescription prevalence per 10,000 registered patients 2001-2009 (95% confidence interval)

	2001	2002	2003	2004	2005	2006	2007	2008	2009
All	3085	3094	3102	3121	3088	3046	3059	3043	3069
analgesics	(3048, 3123)	(3057, 3132)	(3064, 3139)	(3084, 3159)	(3050, 3125)	(3009, 3083)	(3023, 3096)	(3007, 3080)	(3033, 3106)
Basic	1322	1290	1257	1259	1342	1322	1328	1401	1497
analgesics	(1297, 1346)	(1266, 1314)	(1233, 1281)	(1236, 1283)	(1317, 1367)	(1297, 1346)	(1304, 1353)	(1376, 1426)	(1471, 1523)
Topical	272	275	277	306	351	375	399	491	602
NSAIDs	(261, 284)	(264, 287)	(266, 289)	(294, 318)	(338, 364)	(362, 388)	(386, 412)	(476, 505)	(586, 619)
Weak	790	806	813	831	1021	1034	1007	958	905
analgesics	(771, 809)	(787, 825)	(794, 833)	(812, 851)	(999, 1043)	(1012, 1055)	(986, 1028)	(937, 978)	(886, 925)
Moderate	754	715	669	614	393	230	243	250	287
analgesics	(736, 773)	(698, 734)	(651, 686)	(598, 631)	(380, 407)	(220, 241)	(233, 253)	(240, 261)	(276, 298)
Co-proxamol	736	694	652	596	325	124	59	18	11
	(718, 755)	(676, 712)	(635, 669)	(580, 613)	(313, 338)	(116, 131)	(54, 65)	(15, 21)	(9, 14)
Strong	507	536	574	607	752	818	872	909	955
analgesics	(492, 523)	(520, 551)	(558, 591)	(591, 624)	(734, 771)	(799, 837)	(853, 892)	(889, 929)	(935, 976)
Very strong	38	42	41	43	47	50	62	62	74
analgesics	(34, 43)	(37, 46)	(36, 45)	(39, 48)	(42, 51)	(45, 55)	(57, 67)	(57, 68)	(69, 80)
NSAIDs	1146	1187	1264	1301	1187	1121	1091	1011	927
	(1123, 1169)	(1164, 1211)	(1241, 1288)	(1277, 1325)	(1164, 1210)	(1099, 1144)	(1069, 1113)	(990, 1032)	(907, 948)
NSAIDs	1027	988	1009	1005	1067	1027	1029	952	873
excl. Cox-2	(1005, 1049)	(967, 1010)	(988, 1031)	(984, 1027)	(1045, 1089)	(1005, 1048)	(1008, 1050)	(932, 973)	(854, 893)
Cox-2	167	263	317	378	155	116	73	70	65
	(158, 176)	(252, 274)	(305, 329)	(365, 391)	(147, 164)	(109, 124)	(68, 79)	(65, 76)	(60, 71)
