# Supplementary figures and images for: The effectiveness of national guidance in changing analgesic prescribing in primary care from 2002 to 2009: An observational database study
Source: Eur J Pain. 2012 Jul 2;17(3):434–43. doi: 10.1002/j.1532-2149.2012.00189.x (PMC3592995; doi:10.1002/j.1532-2149.2012.00189.x)

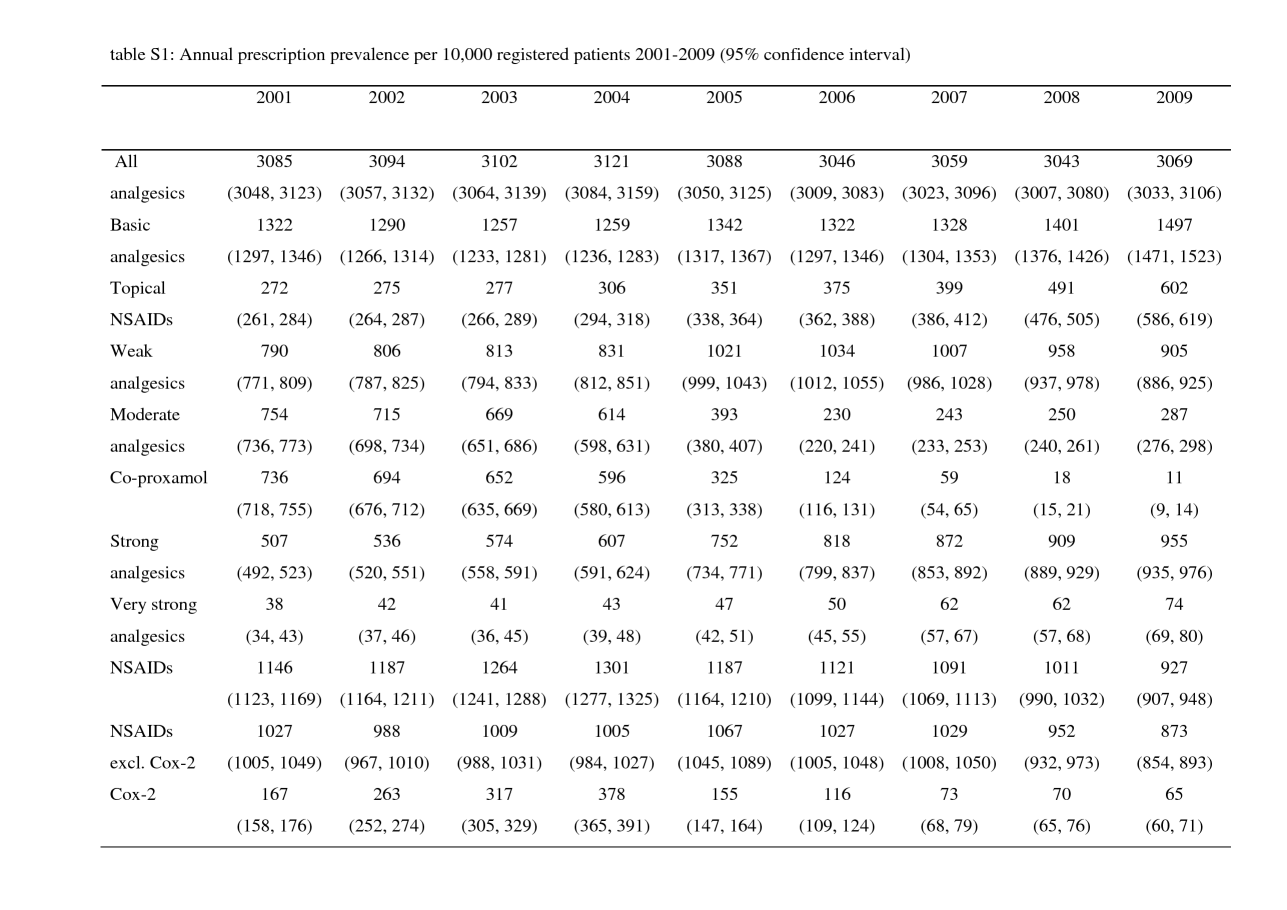

Supplement: Supplementary file 3 [file ejp0017-0434-SD7.png]

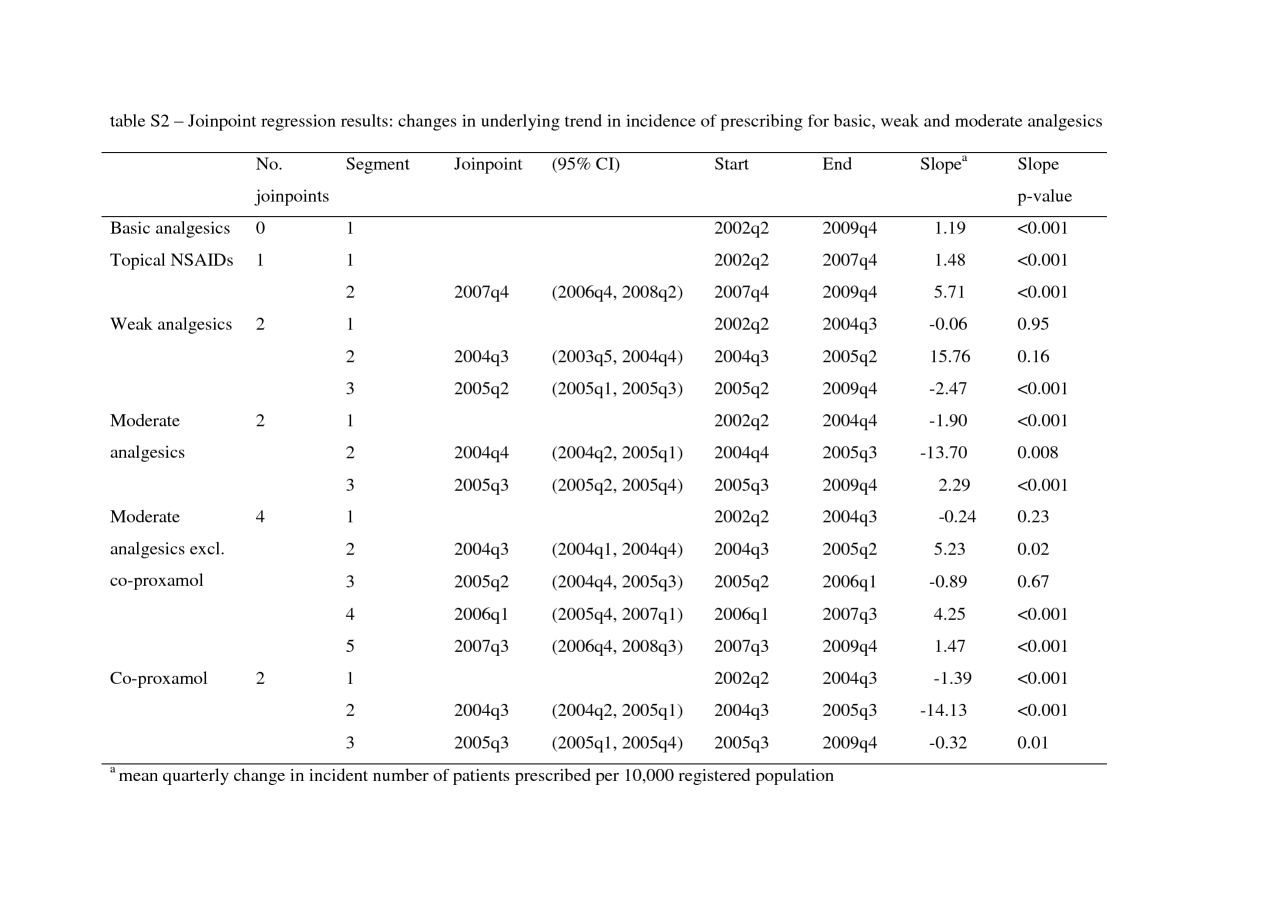

Supplement: Supplementary file 6 [file ejp0017-0434-SD8.png]

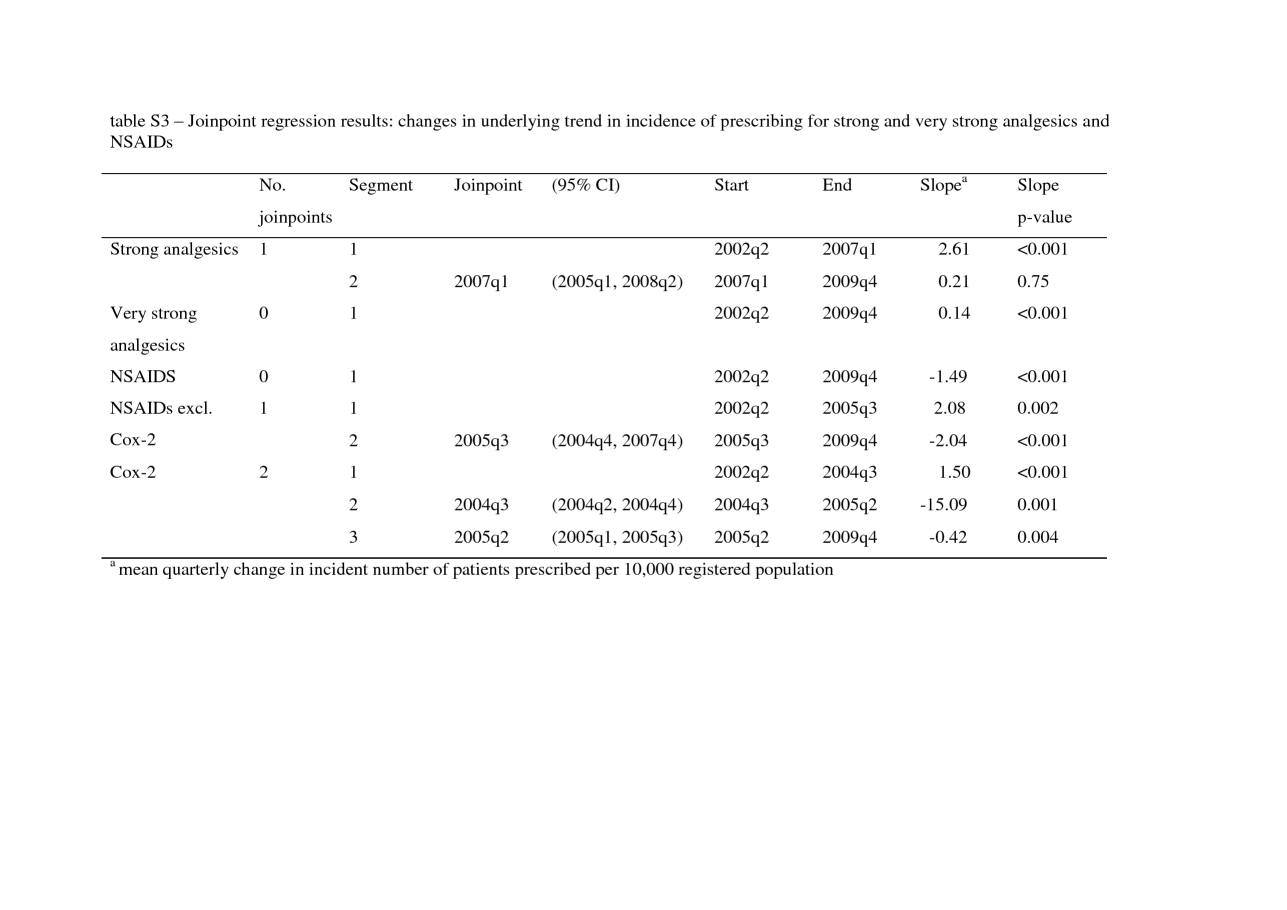

Supplement: Supplementary file 9 [file ejp0017-0434-SD9.png]
