# Supplementary material for: The effectiveness of national guidance in changing analgesic prescribing in primary care from 2002 to 2009: An observational database study
Source: Eur J Pain. 2012 Jul 2;17(3):434–43. doi: 10.1002/j.1532-2149.2012.00189.x (PMC3592995; doi:10.1002/j.1532-2149.2012.00189.x)
Supplement: Supplementary file 4 [file ejp0017-0434-SD2.pdf]

table S2 – Joinpoint regression results: changes in underlying trend in incidence of prescribing for basic, weak and moderate analgesics

	No. joinpoints	Segment	Joinpoint	(95% CI)	Start	End	Slope[a]	Slope p-value
Basic analgesics	0	1			2002q2	2009q4	1.19	<0.001
Topical NSAIDs	1	1			2002q2	2007q4	1.48	<0.001
		2	2007q4	(2006q4, 2008q2)	2007q4	2009q4	5.71	<0.001
Weak analgesics	2	1			2002q2	2004q3	-0.06	0.95
		2	2004q3	(2003q5, 2004q4)	2004q3	2005q2	15.76	0.16
		3	2005q2	(2005q1, 2005q3)	2005q2	2009q4	-2.47	<0.001
Moderate analgesics	2	1			2002q2	2004q4	-1.90	<0.001
		2	2004q4	(2004q2, 2005q1)	2004q4	2005q3	-13.70	0.008
		3	2005q3	(2005q2, 2005q4)	2005q3	2009q4	2.29	<0.001
Moderate analgesics excl. co-proxamol	4	1			2002q2	2004q3	-0.24	0.23
		2	2004q3	(2004q1, 2004q4)	2004q3	2005q2	5.23	0.02
		3	2005q2	(2004q4, 2005q3)	2005q2	2006q1	-0.89	0.67
		4	2006q1	(2005q4, 2007q1)	2006q1	2007q3	4.25	<0.001
		5	2007q3	(2006q4, 2008q3)	2007q3	2009q4	1.47	<0.001
Co-proxamol	2	1			2002q2	2004q3	-1.39	<0.001
		2	2004q3	(2004q2, 2005q1)	2004q3	2005q3	-14.13	<0.001
		3	2005q3	(2005q1, 2005q4)	2005q3	2009q4	-0.32	0.01

[a] mean quarterly change in incident number of patients prescribed per 10,000 registered population
