# Supplementary material for: The effectiveness of national guidance in changing analgesic prescribing in primary care from 2002 to 2009: An observational database study
Source: Eur J Pain. 2012 Jul 2;17(3):434–43. doi: 10.1002/j.1532-2149.2012.00189.x (PMC3592995; doi:10.1002/j.1532-2149.2012.00189.x)
Supplement: Supplementary file 7 [file ejp0017-0434-SD3.pdf]

table S3 – Joinpoint regression results: changes in underlying trend in incidence of prescribing for strong and very strong analgesics and NSAIDs

	No. joinpoints	Segment	Joinpoint	(95% CI)	Start	End	Slope[a]	Slope p-value
Strong analgesics	1	1			2002q2	2007q1	2.61	<0.001
		2	2007q1	(2005q1, 2008q2)	2007q1	2009q4	0.21	0.75
Very strong analgesics	0	1			2002q2	2009q4	0.14	<0.001
NSAIDS	0	1			2002q2	2009q4	-1.49	<0.001
NSAIDs excl. Cox-2	1	1			2002q2	2005q3	2.08	0.002
		2	2005q3	(2004q4, 2007q4)	2005q3	2009q4	-2.04	<0.001
Cox-2	2	1			2002q2	2004q3	1.50	<0.001
		2	2004q3	(2004q2, 2004q4)	2004q3	2005q2	-15.09	0.001
		3	2005q2	(2005q1, 2005q3)	2005q2	2009q4	-0.42	0.004

[a] mean quarterly change in incident number of patients prescribed per 10,000 registered population
